# Man With Something in His Eye

**DOI:** 10.1016/j.acepjo.2025.100146

**Published:** 2025-05-19

**Authors:** Adriel Green, Sameir Alhadi, Ashley Garispe, Martin C. Young

**Affiliations:** Department of Emergency Medicine, Saint Agnes Medical Center, Fresno, California, USA

**Keywords:** fly larvae, maggots, myiasis, osteomyelitis, maggot removal

## 1 Case Presentation

**Figure 1:**
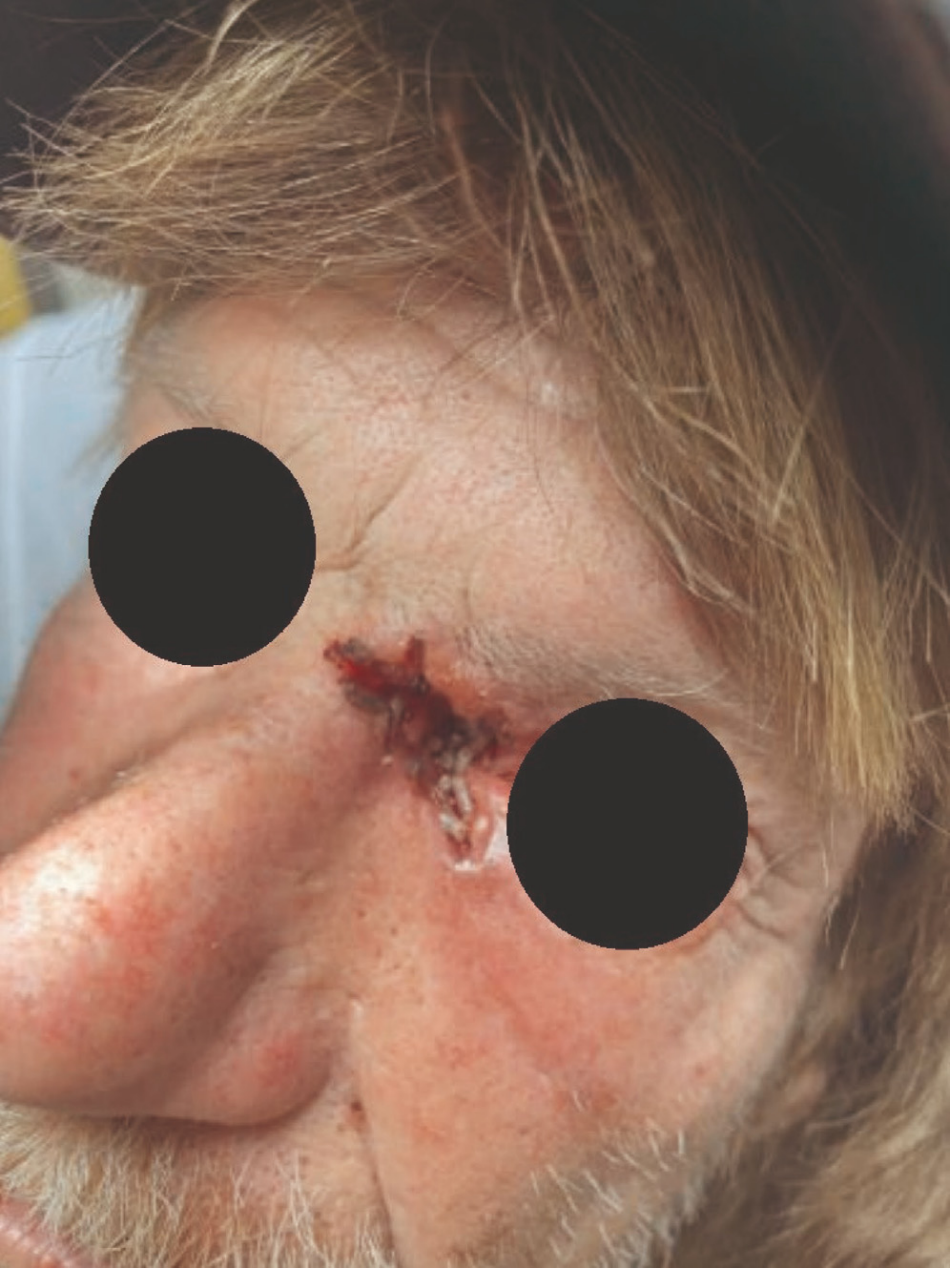
Chronic-appearing wound defect of unknown depth just medial to the left eye with a mass of live insects surrounded by an area of periorbital erythema.

**Figure 2:**
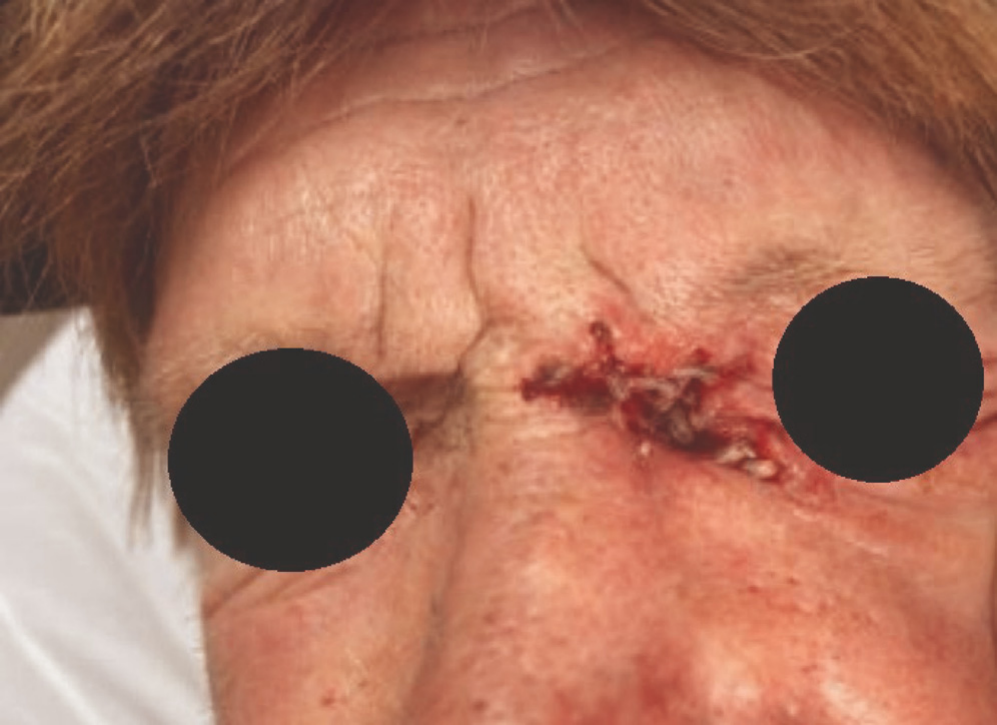
Larvae burrowing deep into the medial left eye.

A 64-year-old Caucasian male with a history of facial basal cell carcinoma status post resection, brain aneurysm, unhoused, and polysubstance drug abuse presented to the emergency department (ED) with left eye pain. Just prior to arrival, he flagged down local rural firefighters seeking transport to the closest hospital. In the ED, he was in significant pain and distress. The physical examination was notable for a chronic-appearing wound defect of unknown depth just medial to the left eye with a mass of live insects surrounded by an area of periorbital erythema (Figs 1 and 2).

## 2 Diagnosis: Facial Myiasis

**Figure 3:**
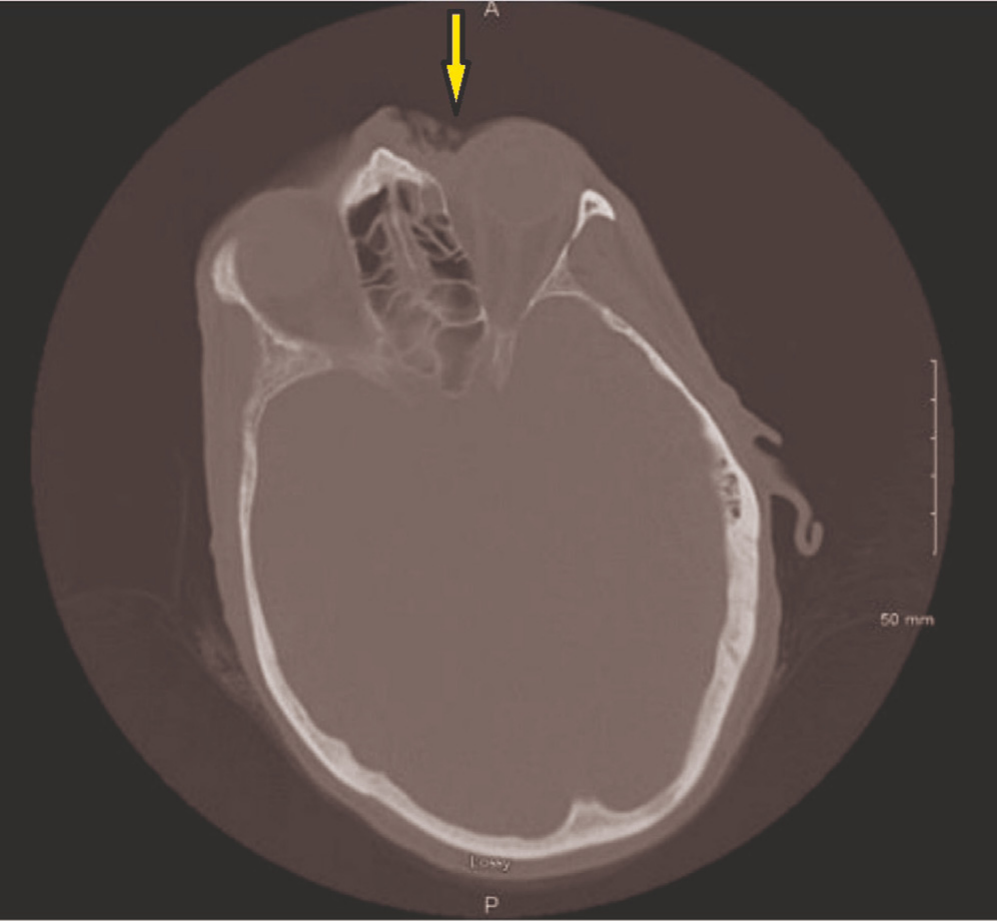
Focal skin ulceration evidenced on computer tomography (yellow arrow).

**Figure 4:**
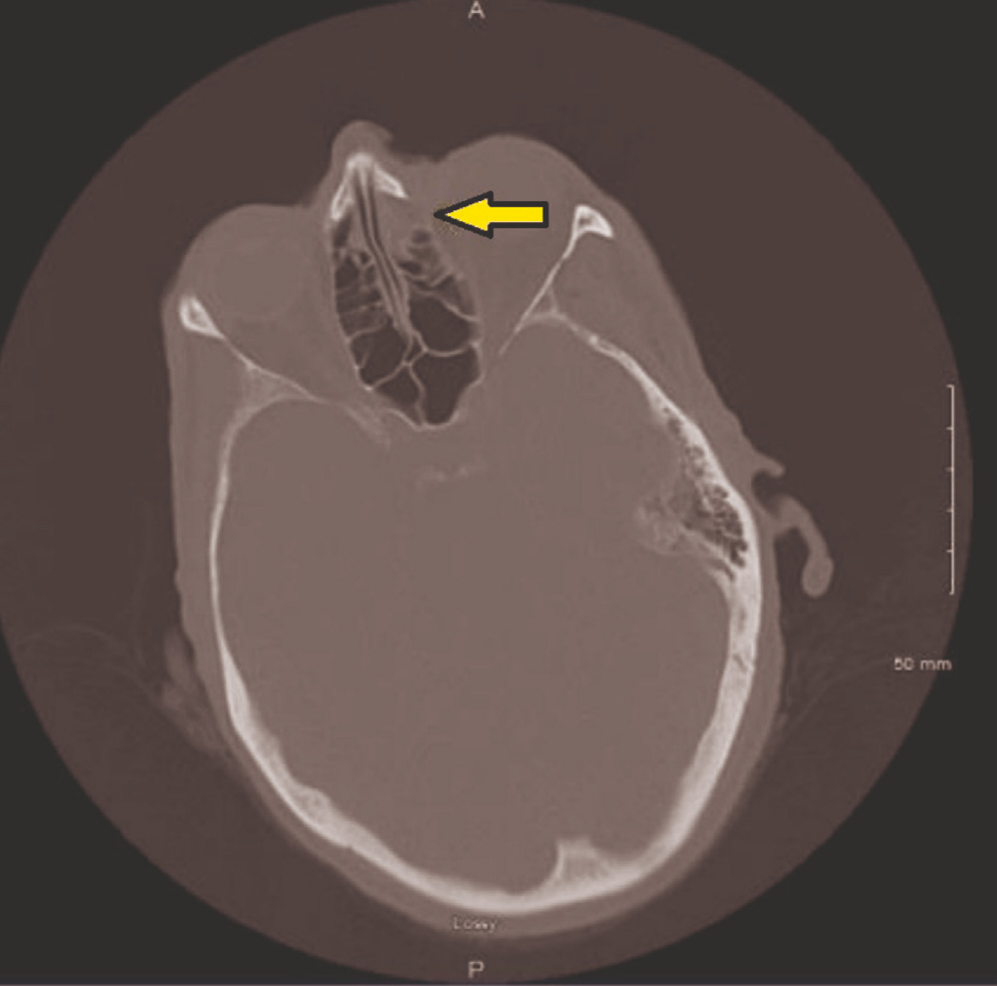
Erosion into the ethmoid sinus (yellow arrow).

Facial myiasis (parasitic infection of maggot infestation) with concomitant osteomyelitis was demonstrated on computer tomography maxillary or face via ulceration along the left nasal canthus and nasal soft tissues with focal erosions of the left anterior ethmoid sinus (Figs 3 and 4). Unasyn and vancomycin were started with admission to the medicine service. Oral and maxillofacial surgery, otolaryngology, and ophthalmology were consulted.

Further investigation revealed his basal cell carcinoma was treated with surgical resection 2 years prior; however, due to social barriers to health, including unhoused status and lack of reliable transportation, he was lost to follow-up with oncology. In the interim, he has undergone multiple cycles of scabbing and unroofing, recently reporting worsening pain, purulent discharge, and a sensation of movement in his face.

After approximately 24 hours of being admitted to the floor, he refused vitals, telemetry, and antibiotics. The patient became agitated and aggressive and left against medical advice prior to specialty evaluation.

Thirteen months later, the same patient presented to the ED with worsening left eye pain and purulent drainage but no maggots. The ED physician consulted with oncology, the oral and maxillofacial surgery chair, and social services to discharge him and send him by taxi to the local tertiary center. No records are available after this encounter.

Facial myiasis is a rare but notable phenomenon with diverse etiologies. Although myiasis commonly occurs in tropical regions and among individuals with poor hygiene and compromised immune systems, concomitant malignancy cases presenting in more temperate climates are infrequent and often pose diagnostic and therapeutic challenges.^1^

No standard protocol exists for the treatment of myiasis, thus allowing different therapies to be used depending on the situation and circumstance.^1^ Treatment options in the ED include mechanical larvae removal, suffocation, and starting pharmacotherapy, such as broad-spectrum antibiotics and ivermectin.^2^, ^3^, ^4^ Mechanical removal may benefit from local anesthetic preparations to minimize discomfort from the use of forceps and copious irrigation. If the larvae are burrowed deep into the tissue/wound and accessing them becomes a challenge, the method of suffocation using noninvasive approaches of placing petroleum jelly, liquid paraffin, beeswax, heavy oil, or even bacon strips over the wound may help to facilitate the migration of the maggots closer to the skin surface.^3^^,^^4^ The choice of antibiotics should be individually risk-stratified to cover for possible cellulitis vs osteomyelitis, depending on the examination findings. Ivermectin may show promise due to its inherent properties of binding selectively with glutamate-gated chloride ion channels in invertebrate nerve and muscle cells, causing cell death.^2^^,^^4^ If the larvae invasion does not respond to treatment or poses other health risks, surgical debridement may be necessary.^1^

## 3 Key Points

1. Facial myiasis generally affects individuals with poor hygiene habits, drug users, and psychosocial disorders but is often associated with malignant tumors.
2. Larvae removal may require ED creativity with the use of suffocation methods.
3. Ivermectin may target the invasive invertebrate maggots, but do not forget to cover for concomitant skin and/or bone infections with the appropriate antibiotics.

## Funding and Support

By *JACEP Open* policy, all authors are required to disclose any and all commercial, financial, and other relationships in any way related to the subject of this article as per ICMJE conflict of interest guidelines (see www.icmje.org). The authors have stated that no such relationships exist.
